# Sex differences in hearing impairment due to diet-induced obesity in CBA/Ca mice

**DOI:** 10.1186/s13293-023-00493-z

**Published:** 2023-02-21

**Authors:** Soo Jeong Kim, Akanksha Gajbhiye, Ah-Ra Lyu, Tae Hwan Kim, Sun-Ae Shin, Hyuk Chan Kwon, Yong-Ho Park, Min Jung Park

**Affiliations:** 1 Brain Research Institute, College of Medicine, Chungnam National University, 282 Munwha-ro, Daesa-dong, Jung-gu, 35015 Daejeon, South Korea; 2 Department of Medical Science, College of Medicine, Chungnam National University, Daejeon, 35015 South Korea; 3 Department of Otolaryngology-Head and Neck Surgery, College of Medicine, Chungnam National University, Daejeon, 35015 South Korea

**Keywords:** Hearing loss, HFD-induced obesity, Auditory brainstem response (ABR), Distortion product otoacoustic emissions (DPOAE), Wave 1 amplitude, CtBP2, Ribbon synapse, Sex differences, Heme oxygenase-1 (HO-1), HSP 32, Total adiponectin, Adiponectin receptor 1, G3BP1, Stress granule

## Abstract

**Background:**

Obesity is an independent risk factor for hearing loss. Although attention has focused on major obesity comorbidities such as cardiovascular disease, stroke, and type 2 diabetes, the impact of obesity on sensorineural organs, including the auditory system, is unclear. Using a high-fat diet (HFD)-induced obese mouse model, we investigated the impact of diet-induced obesity on sexual dimorphism in metabolic alterations and hearing sensitivity.

**Methods:**

Male and female CBA/Ca mice were randomly assigned to three diet groups and fed, from weaning (at 28 days) to 14 weeks of age, a sucrose-matched control diet (10 kcal% fat content diet), or one of two HFDs (45 or 60 kcal% fat content diets). Auditory sensitivity was evaluated based on the auditory brainstem response (ABR), distortion product otoacoustic emission (DPOAE), and ABR wave 1 amplitude at 14 weeks of age, followed by biochemical analyses.

**Results:**

We found significant sexual dimorphism in HFD-induced metabolic alterations and obesity-related hearing loss. Male mice exhibited greater weight gain, hyperglycemia, increased ABR thresholds at low frequencies, elevated DPOAE, and lower ABR wave 1 amplitude compared to female mice. The hair cell (HC) ribbon synapse (CtBP2) puncta showed significant sex differences. The serum concentration of adiponectin, an otoprotective adipokine, was significantly higher in female than in male mice; cochlear adiponectin levels were elevated by HFD in female but not male mice. Adiponectin receptor 1 (AdipoR1) was widely expressed in the inner ear, and cochlear AdipoR1 protein levels were increased by HFD, in female but not male mice. Stress granules (G3BP1) were significantly induced by the HFD in both sexes; conversely, inflammatory (IL-1β) responses were observed only in the male liver and cochlea, consistent with phenotype HFD-induced obesity.

**Conclusions:**

Female mice are more resistant to the negative effects of an HFD on body weight, metabolism, and hearing. Females showed increased peripheral and intra-cochlear adiponectin and AdipoR1 levels, and HC ribbon synapses. These changes may mediate resistance to HFD-induced hearing loss seen in female mice.

## Highlights

- Females are protected against HFD-induced metabolic alterations and hearing impairment.
- Sex differences were seen in the ABR wave 1 amplitude, which correlated with loss of hair-cell ribbon synapses (CtBP2).
- The serum adiponectin concentration was significantly higher in female compared to male mice, and the cochlear adiponectin levels were elevated by HFD in females, but not male mice.
- Cochlear and liver AdipoR1 expression evidenced significant sex differences in CBA/Ca mice.
- The core stress granule component, G3BP1, was induced by HFD in the cochleae of male and female mice. Significant sex differences were found in cochlear blood flow and IL-1β concentration.

## Background

Obesity is a serious and chronic condition caused by a positive energy balance that impacts physiological and psychological quality of life. Although attention has focused on major obesity comorbidities such as cardiovascular disease, stroke, and type 2 diabetes [1], the impact of obesity on sensorineural organs, such as the auditory system, is unclear. Obesity is associated with hearing loss, and higher waist circumference and body mass index (BMI) are associated with the severity of hearing loss [2, 3]. Obesity-related metabolic disorders in an animal model of type 2 diabetes are associated with hearing impairment [4–8]. Having a hearing deficit, in addition to obesity and its comorbidities, is devastating because communication is impaired, which hampers social inclusion and exacerbates obesity.

Adipose tissue secretes bioactive molecules such as adipokines including leptin, adiponectin, resistin, and adipsin [9]. Adiponectin is the most abundant adipokine and protects against various obesity-related morbidities, including hypertension, hyperlipidemia, and type 2 diabetes [9, 10]. Hwang et al. reported a protective role for adiponectin against age-related hearing loss in a clinical cohort [11]. Wu et al. performed an investigation of the adiponectin gene using single-nucleotide polymorphisms [12], and demonstrated antiapoptotic effects of adiponectin in cochlear explant cultures [12]. These data suggest that adiponectin functions not only as a modulator of obesity-related vascular and metabolic disorders, but also as an otoprotectant against hearing loss.

Sex, as an important biological variable, affects obesity-related metabolic outcomes and auditory sensitivity. Pettersson et al*.* found that male mice are more vulnerable than females to the effects of a high-fat diet (HFD) on body weight and metabolism [13]. Additionally, young [14] and old [15, 16] male mice reportedly suffer more hearing loss than female mice. Sexual dimorphism is found in metabolism and hearing sensitivity; however, few preclinical studies have investigated sex-based differences in obesity-related hearing loss. In this study, we investigated the impact of diet-induced obesity (DIO) on sex differences in hearing sensitivity and metabolic changes in CBA/Ca mice.

## Methods

### Ethics statement

All animal experiments were approved by Chungnam National University Hospital (CNUH), Institutional Animal Care and Use Committee (IACUC, reference numbers: CNUH-020-A0007, CNUH-021-P0017, and CNUH-2022-IA0084-00). All animal care and use was conducted in accordance with the Guide for the Care and Use of Laboratory Animals.

### Animals and diets

Male and female CBA/Ca mice (Envigo, The Netherlands) were used in this study (a total of 54 animals). Maintenance diet was chow (2018 Teklad global 18% protein rodent diets, Envigo) until weaning at 28 days of age, after which mice were allowed to continue on sucrose matched 10-(Research Diets Inc. # D12450J), 45-(Research Diets Inc. #D19102806), or 60-(Research Diets Inc. # D12492) kcal % fat diets ad libitum. Mice were randomly assigned to these given groups for further studies. Body weight and fasting blood glucose levels (Accu-Chek Performa, Roche) were recorded biweekly from 4 weeks of age to 14 weeks of age.

### Auditory brainstem response (ABR) and distortion product otoacoustic emissions (DPOAE)

All auditory tests were conducted in a sound-attenuated booth (Sontek, South Korea) on mice anesthetized with intramuscular injection of zolazepam HCl 40 mg/kg (Zoletil, Virbac Animal Health, Carros, France) and xylazine 10 mg/kg (Rompun, Bayer Animal Health, Monheim, Germany). The TDT System-3 (Tucker Davis Technologies, Gainesville, FL, USA) hardware (RZ6 Processor) and software (BioSig RP, ver. 4.4.1; Tucker Davis Technologies) were used to obtain the ABR and DPOAE. ABR thresholds at frequencies between 4 and 32 kHz, and click sounds, were obtained separately from both ears. The stimuli were computer-generated tone pips. Subcutaneous needle electrodes were placed around the skull vertex and both infraauricular areas. Tone bursts, with a duration of 4 ms and rise–fall time of 1 ms at frequencies of 4, 8, 16, and 32 kHz, were used, in addition to click. The sound intensity was varied in 5-dB decrements. The contralateral ear was not masked because the stimuli were transmitted through a sealed earphone. The waveforms were analyzed using a custom program (BioSig RP, ver. 4.4.1; Tucker Davis Technologies) with the researcher blinded to the treatment group. Threshold was defined as the lowest stimulus intensity to evoke a wave III response > 0.2 μV.

For DPOAE tests, a single acoustic assembly consisting of an ER-10B + (Etymotic, Elk Grove Village, IL, USA) connected to two TDT MF-1 transducers was inserted into the external ear canal such that an unobstructed path from port to tympanic membrane was established with an appropriate acoustic seal. Two primary tones consisting of *L*1 = 65 dB SPL and *L*2 = 55 dB SPL were presented at *f*2 frequencies ranging 0.545–35.344 kHz (*f*2/*f*1 = 1.2). The amplitude of the DPOAE at 2*f*1 − *f*2 was recorded with background noise estimates at 6 surrounding spectra. Immediately following DPOAE recordings, a closed-field TDT MF-1 speaker was placed in the left ear of each mouse, and subdermal needle electrodes (Rhythmlink, Columbia, SC, USA) were placed at the vertex (noninverting), under the test ear (inverting), and at the base of the tail (ground). Tone-burst stimuli (Blackman window, 3 ms, 1.5 ms rise/fall in alternating polarity) were presented at a rate of 29.9/s at 4, 8, 16, and 32 kHz starting at 90 dB SPL. The waveforms from 1024 presentations were averaged, amplified (20 ×), filtered (0.3–3 kHz) and digitized (25 kHz). Stimulus level decreased in 20-dB steps and in then in 5-dB steps near threshold. Auditory threshold was defined as the lowest stimulus level that elicited a reproducible waveform with identifiable peaks.

### Cochlear dissection and immunostaining

Samples from male and female mice aged 14 weeks were collected. Cochlear tissues were obtained and fixed in 4% paraformaldehyde in phosphate-buffered saline (PBS) for 30 min at room temperature as previously described [4, 17]. The separated individual cochlear turns were prepared for whole-mount immunostaining.

For whole-mount immunostaining, dissected cochleae were blocked in 0.3% Triton X-100 (Sigma-Aldrich, St. Louis, MO, USA) in 5% normal goat serum (Vector Laboratories, Burlingame, CA, USA) for 1 h, then incubated with primary antibody (antibody list, Table 1) in blocking solution overnight at 4 °C (Hoechst 33342 for 1 min). After rinsing six times in PBS for 10 min, the tissues were incubated with each secondary antibody in PBS for 2 h (antibody list, Table 1). The specimens were mounted on glass slides using CrystalMount (Biomeda, Foster City, CA, USA) and observed under an epifluorescence microscope (Zeiss Axio Scope A1, Zeiss, Oberkochen, Germany) with a digital camera.

**Table 1:** Antibody list

Antibody	Company	Catalog #	Dilution
Adiponectin	Abcam	ab22554	1:200
Adiponectin/Acrp30 Antibody [Alexa Fluor® 488] (0.1 ML)	NOVUS	NB100-65810AF488	1:200
AdipoR1	Santa Cruz	SC-518030	1:1000
AdipoR1 (D-9) Alexa Fluor® 594	Santa Cruz	SC-518030 AF594	1:200
β-actin	Cell signaling	4967s	1:3000
COX IV	Cell signaling	11967	1:1000
CtBP2	BD Bioscience	Cat#612044	1:200
G3BP1	Cell signaling	69101	1:1000
HMOX1 (HO-1, HSP 32)	Invitrogen	PA5-27338	1:1000
Hoechst 33342	Invitrogen	H3570	1:1000
MyoVIIa	Proteus	25-6790	1:200
PSD-95	Abcam	ab18258	1:200
Alexa Fluor 594 Phalloidin (F-actin probe)	Invitrogen	A12381	1:500
Alexa Fluor 594, Goat anti-Rabbit IgG (H+L) Cross-Adsorbed ReadyProbes™ Secondary Antibody	Invitrogen	R37117	1:500
Alexa Fluor 594, Goat anti-Rabbit IgG (H+L) Highly Cross-Adsorbed Secondary Antibody	Invitrogen	A11037	1:500
Alexa Fluor 488, Goat anti-Rabbit IgG (H+L) Highly Cross-Adsorbed Secondary Antibody	Invitrogen	A11034	1:500
Alexa Fluor 488, Goat anti-Mouse IgG (H+L) Cross-Adsorbed Secondary Antibody	Invitrogen	A11001	1:500
Alexa Fluor 488, Goat anti-Chicken IgY (H+L) Secondary Antibody	Invitrogen	A11039	1:500

For section immunostaining, 4% PFA-fixed, and 120 mM EDTA-decalcified, paraffin-embedded tissues were prepared and stored at − 20 °C prior to sectioning at 4 µm thickness. Blocking and antibody hybridization steps were carried out in PBS containing 1.5% normal chicken serum and 0.3% Triton X-100. Section immunostaining was observed by epifluorescence microscopy (BX53F2, Olympus, Tokyo, Japan).

### Protein extraction and quantification

Tissue samples were collected and homogenized in lysis buffer (RIPA Lysis and Extraction Buffer, Thermo Scientific, # 89900) with Halt™ protease and phosphatase inhibitor cocktail (Thermo Scientific, # 78442), and lysates were collected after centrifugation at 20,000*g* for 30 min. Protein concentrations were determined using the BCA protein assay kit (Thermo Scientific, #23225) and the plates were read at 562 nm in a microplate reader (Tecan US Inc., Durham, NC, USA).

### Western blotting

Approximately 5–10 µg of protein were separated by 10% SDS–polyacrylamide gel electrophoresis and electroblotted onto polyvinylidene difluoride (PVDF) membranes (Millipore, Burlington, MA, USA). After blockage of nonspecific binding sites, the membranes were incubated with a primary antibody (Table 1) overnight at 4 °C. The membranes were then washed with PBS and incubated with a secondary antibody (Table 1) at room temperature for 2 h. Blots were visualized using Immobilon Western Chemiluminescent HRP Substrate (Millipore, Burlington, MA, USA) and the images were acquired and quantitated using Azure 300 Chemiluminescent Western Blot Imaging System (Azure Biosystems, Dublin, CA, USA).

### Measurement of tissue levels of total adiponectin, leptin and IL-1β

Serum and cochlear adipokines were measured using standard sandwich enzyme-linked immuno-sorbent assay mouse total adiponectin (R&D Systems, Minneapolis, MN, #MRP300), leptin (Abcam, Boston, MA, #ab100718), and IL-1β (R&D Systems, Minneapolis, MN, #MLB00C) ELISA Kits as per the manufacturer’s instruction. Samples, standards, and controls were added to appropriate wells in a 96-well plate, as stated in the kit protocol, and incubated for 3 h at room temperature. After washing, 50 μL of conjugate was added to each well and incubated at room temperature for 1 h on the shaker. After washing and incubation in substrate solution for 30 min, the plates were read at 450 nm in a microplate reader (Tecan US Inc., Durham, NC, USA). Sample measurements were interpolated from the standard curve, and values from tissue lysates were normalized to total protein concentrations.

### Measurement of cochlear blood flow

The left tympanic bulla of each mouse was exposed and opened under anesthesia as described previously [17]. Briefly, the cochlear blood flow was measured at 14 weeks of age while the mouse was placed on the stereotaxic instrument, using a 0.1-mm-diameter laser Doppler probe connected to a Laser Doppler Flowmeter (Transonic Systems, Ithaca, NY, USA). Cochlear blood flow was determined from an intensity oscillation that was translated from the frequency of the oscillation.

### Image processing and statistical analysis

Two-way ANOVA coded for sex and diet was used, followed by planned comparisons. Specific statistical tests are noted in respective figure legends. Data normality was tested by the D’Agostino–Pearson omnibus normality test when sample size was sufficient to do so. All tests were performed using Prism GraphPad Version 9.4.1. Group differences were considered significant at p < 0.05 in each case. Adobe Photoshop CS6 was used for adjustment of image contrast, colorization of monochrome fluorescence images, and superimposition of images.

## Results

### Female mice are protected against diet-induced obesity

Mice were subjected to hearing tests prior to HFD feeding; the auditory brainstem response (ABR) and distortion product otoacoustic emission (DPOAE) were used to evaluate baseline hearing sensitivity and outer hair cell (OHC) function at 4 weeks of age. No significant outliers or deficits in ABR and DPOAE were found, so all tested animals were included in the subsequent experiments. CBA/Ca mice were used because their hearing is stable over time, whereas C57BL/6 mice suffer a steady, early decline of hearing due to a recessively inherited mutation in *Cdh23*, which underlies the *Ahl* locus [18, 19]. We evaluated two high-fat diets of 45% and 60% w/w fat. We included a 45% fat diet in addition to the “traditional” 60% diet because a rodent diet with a 45% fat content may be more relevant to human physiology than a 60% fat diet; the typical American or European diet contains about 36–40% fat by energy [20].

**Fig. 1:**
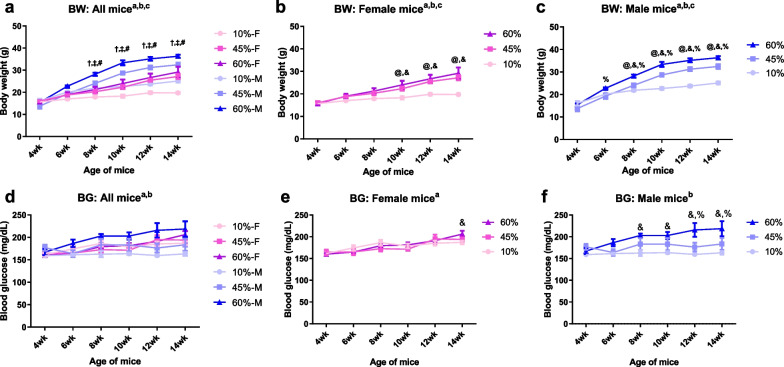
Female mice are protected against diet-induced obesity. **a**–**c** Biweekly body weight change of CBA/Ca male and female mice fed with a control diet (low-fat diet, 10 kcal% fat content diets), or two high-fat diets (HFD, 45 kcal% or 60 kcal% fat content diets). All mice responded to HFDs compared to control diet. The HFD-induced weight gains appeared at an earlier time (**b**, **c**) and were much greater (**a**) in the males as compared with the females. Two-way ANOVA. Tukey's multiple comparisons test. ^a^Main effect of age; ^b^main effect of diet; ^c^interaction; †, 10% male vs. female; ‡, 45% male vs. female; #, 60% male vs. female; @, 10% vs. 45%; &, 10% vs. 60%; %, 45% vs. 60%. **d**–**f** Fasting blood glucose measured biweekly from both female and male CBA/Ca mice. The obese male mice, but not the females, developed significant hyperglycemia. HFD feeding significantly elevated a fasting blood glucose level in male mice. Two-way ANOVA. Tukey's multiple comparisons test. a, main effect of age; ^b^main effect of diet; @, 10% vs. 45%; and, 10% vs. 60%; %, 45% vs. 60%. n = 6

Male and female mice were randomly assigned to one of three diet groups for 12 weeks: control diet (low-fat diet, 10 kcal% fat content) or one of two HFD (45 or 60 kcal% fat content). HFD-fed mice showed increased body weight compared to the control group fed a standard (10 kcal% fat content) diet (Fig. 1a). HFD-induced weight gains appeared earlier (Fig. 1b–c) and were greater in male compared to female mice. In male mice, both types of HFD caused a significant increase in body weight, starting from 6 weeks of age, and was maintained until the end of the experiment (Fig. 1c). Female mice exhibited a smaller increase in body weight after 10 weeks of age (Fig. 1b).

Fasting blood glucose levels were measured biweekly to assess metabolic alterations induced by the HFD (Fig. 1d). Obese male (Fig. 1f), but not female (Fig. 1e), mice developed significant hyperglycemia. Therefore, HFD feeding increased the fasting blood glucose level in male mice.

### Male mice are more vulnerable to HFD-induced hearing loss

**Fig. 2:**
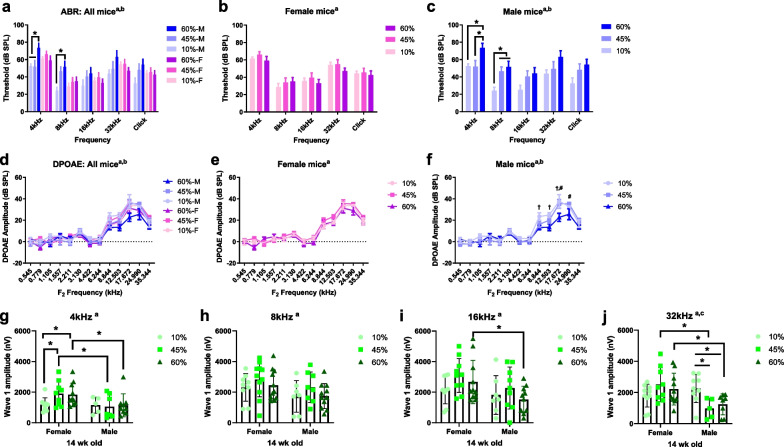
Male mice are more vulnerable to HFD-induced hearing loss. **a** Auditory sensitivity was evaluated by auditory brainstem response (ABR) threshold in all mice (**a**) at 14 weeks of age (10 weeks of diet treatments). Two-way ANOVA. ^a^main effect of frequency (*F*_(4, 222)_ = 21.24, *p* < 0.0001); ^b^main effect of diet (*F*_(5, 222)_ = 6.941, *p* < 0.0001). **b** Female mice showed no significant differences in ABR threshold between diet groups. ^a^Main effect of frequency. **c** HFD-fed (60 kcal% fat) male mice presented significantly decreased hearing sensitivity (increased ABR threshold) at 4- and 8-kHz frequency compared to other diet groups. ^a^main effect of frequency; b, main effect of diet, *p* < 0.05. *n* = 6–10. **d** Distortion product otoacoustic emissions (DPOAE), an indirect measure of outer hair cell (OHC) function, were recorded from mice in all treatment groups at 14 weeks of age. DIO impaired outer hair cell function in males, but not in females. **e** Female mice had no significant reduction in DPOAE amplitudes in all treatment groups relative to baseline (*F*_(2,351)_ = 5.145, *p* = 0.0063; *p* > 0.05 for all multiple comparisons). **f** HFD-fed (60 kcal% fat) male mice impairs outer hair cell function. Two-way ANOVA. ^a^Main effect of frequency, *F*_(12, 273)_ = 51.29, *p* < 0.0001; ^b^main effect of diet, *F*_(2, 273)_ = 7.707, *p* = 0.0006; ^c^interaction, *F*_(24, 273)_ = 1.436, *p* = 0.0892; †, 10% vs. 60%; #, 45% vs. 60%. **g**–**i** Sex differences in auditory nerve function. ABR wave I amplitudes (a sensitive measure of auditory nerve function) were elicited by tone pips (90 dB) at 4 (**g**), 8 (**h**), 16 (**i**), and 32 (**j**) kHz at 14 weeks of age. *n* = 8–11. Asterisk indicates *p* < 0.05. ^a^main effect of sex; ^b^main effect of diet; Two-way ANOVA; Tukey's multiple comparisons test

Auditory sensitivity was evaluated based on the ABR threshold (Fig. 2a) at 14 weeks of age (10 weeks on the diet). In female mice (Fig. 2b), no significant difference in ABR threshold was seen according to diet group. Conversely, 60 kcal% HFD-fed male mice (Fig. 2c) had an increased ABR threshold at the 4 and 8 kHz frequencies compared to the control, indicating significantly decreased hearing sensitivity among male mice.

DPOAE, as an indirect measure of OHC function, was recorded at 14 weeks of age. DIO impaired OHC function in males, but not females (Fig. 2d–f). Female mice exhibited no significant reduction in DPOAE relative to baseline, regardless of treatment group (Fig. 2e). HFD-fed male mice showed reduced DPOAEs (Fig. 2f), suggesting dysfunctional OHCs and/or changes in the endocochlear potential followed by stria vascularis damage in this group.

Next, ABR wave I amplitudes, as a measure of auditory nerve function, were elicited by tone pips (90 dB) at 4 (Fig. 2g), 8 (Fig. 2h), 16 (Fig. 2i), and 32 (Fig. 2j) kHz at 14 weeks of age. At 4 kHz, the wave 1 amplitude was significantly improved by HFDs in female, but not male, mice compared to control diet-fed mice (female 10% vs*.* 45%, *p* = 0.0332; female 10% vs. 60%, *p* = 0.0494; male 10% vs. 45%, *p* = 0.8225; male 10% vs. 60%, *p* = 9873). Moreover, a significant main effect of sex was observed when comparing the wave 1 amplitude of HFD-treated female mice to that of males at 4 kHz (*F*_1, 43_ = 6.528, *p* = 0.0142, Fig. 2g), 8 kHz (*F*_1, 51_ = 5.224, *p* = 0.0265, Fig. 2h), 16 kHz (*F*_1, 50_ = 5.832, *p* = 0.0194, Fig. 2i), and 32 kHz (*F*_1, 45_ = 7.332, *p* = 0.0095, Fig. 2j), indicating decreased auditory nerve function of male compared to female mice. Specifically, at 32 kHz, the wave 1 amplitude of HFD-fed (45% and 60%) male mice was significantly decreased compared to that of control diet-fed males (male 10% vs. 45%, *p* = 0.0129; male 10% vs. 60%, *p* = 0.0232), suggesting that synaptic transmission was affected by the HFD in male mice. Overall, these data indicate that male mice are more susceptible to HFD-related metabolic alterations and hearing loss.

### Effect of HFD on HC loss

**Fig. 3:**
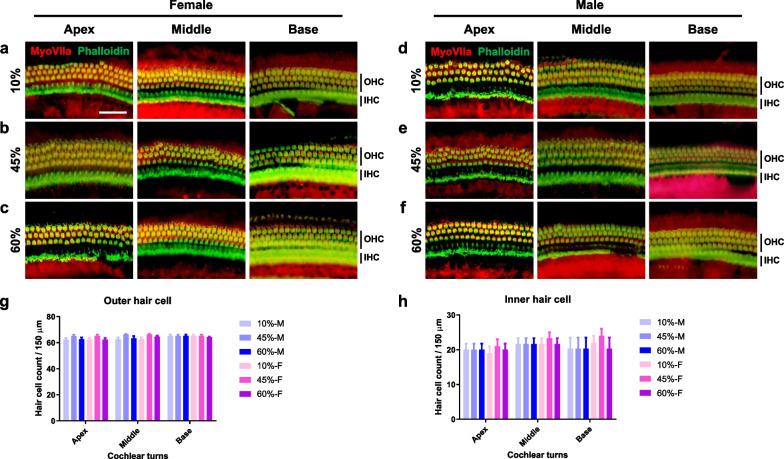
Cochlear hair cells are intact throughout all treatment groups. Whole-mount preparations of the auditory epithelium in the female (**a**–**c**) and male (**d**–**f**) mice. Tissues were stained for myosin VIIa (red, hair cell marker) and phalloidin (green, filamentous actin, F-actin, marker), then photographed using epifluorescence. No hair cell loss was observed on IHCs and OHCs: apex, middle, and basal turns. Scale bar = 50 μm. **g**, **h** OHCs (**g**) and IHCs (**h**) remained intact in all cochlear turns. Two-way ANOVA; Tukey's multiple comparisons test; all comparisons, *p* > 0.05

To investigate the pathophysiology of hearing loss in male mice after control or HFD treatment, whole-mount preparations of the auditory epithelium were stained for myosin VIIa (hair cell [HC] marker) and phalloidin (filamentous actin marker), and photographed using an epifluorescence microscope. In female (Fig. 3a–c) and male (Fig. 3d–f) mice, no inner hair cell (IHCs) loss, or loss of OHCs of the apex, middle, and basal turns, was seen in any diet group (Fig. 3g, h). Therefore, cochlear HCs are not affected by diet or sex.

### Sex differences in pre-synaptic ribbons on IHC synapses

**Fig. 4:**
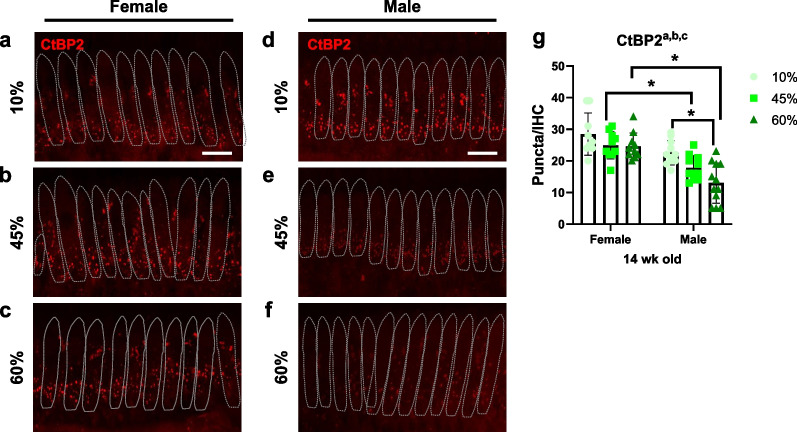
Density of HC ribbon synapses (CtBP2) are sexually dimorphic. **a**–**f** Confocal images of CtBP2 (red) puncta in the IHC synapses of female (**a**–**c**) and male (**d**–**f**) mice (30 kHz region). **g** The number of pre-synaptic ribbon synapses were examined from whole-mount cochleae. Quantified analysis confirmed that sex differences in the expression of CtBP2 puncta were found in the inner hair cells and synapses. Two-way ANOVA; Tukey's multiple comparisons test; *p < 0.05. Scale bar = 10 μm

Our data indicate that male mice are more vulnerable to obesity-related hearing impairment. However, the hearing deficit and sex difference were not explained by differential loss of IHCs and OHCs in the inner ear (Figs. 2, 3). Cochlear synaptopathy, i.e., loss of IHC functional synapses, accounts for the reduction in wave 1 amplitude seen following noise exposure and with aging [21]. Therefore, the pre-synaptic ribbons were immunostained for C-terminal binding protein 2 (CtBP2). Confocal images (Z-stacks) of CtBP2 (red) puncta were obtained from whole-mount cochleae (Fig. 4a–f), and the pre-synaptic ribbons were quantified (Fig. 4g). There was no significant difference in pre-synaptic ribbons between low-fat diet (LFD)-fed male and female mice (10% male vs. female, *p* = 0.1063). However, a significant decrease in the number of pre-synaptic ribbons per IHC was observed in the HFD-fed compared to LFD-fed male mice (10% vs*.* 60%, *p* = 0.0004), and in HFD-fed females compared to males (45% female vs*.* male, *p* = 0.0227; 60% female vs*.* male, *p* < 0.0001). Interestingly, no difference was detected between LFD-fed female and HFD-fed female mice (*p* > 0.05, two-way ANOVA with Tukey’s multiple comparisons test). For CtBP2, there was a main effect of sex (*F*_1, 57_ = 42.15, *p* < 0.0001), suggesting that an HFD induces more severe synaptopathy in the cochlea of male compared to female mice. Therefore, the change in pre-synaptic ribbons may account for the sex differences in the effects of HFD-induced obesity on hearing.

### Sexual dimorphism in total levels of adiponectin

**Fig. 5:**
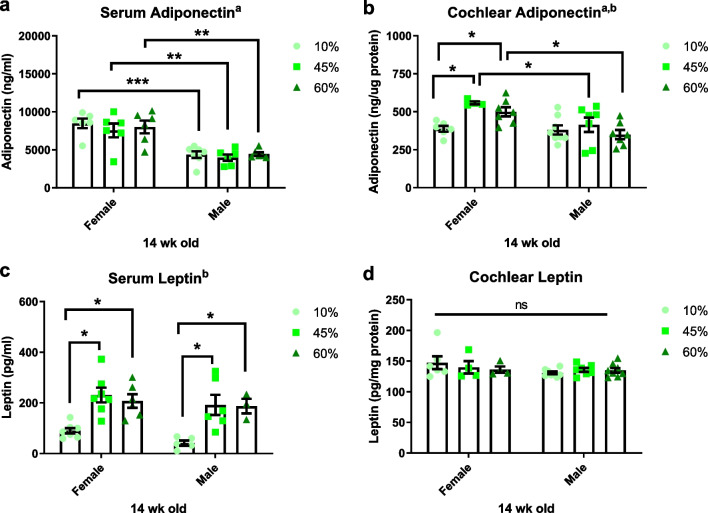
Total adiponectin expression shows sex differences; leptin is regulated by diet but not sex. **a** Serum/circulating adiponectin levels were significantly higher in females compared to males (^a^main effect of sex, *F*_(1, 31)_ = 55.00, *p* < 0.0001), however, there was no main effect of diet treatments (*F*_(2, 31)_ = 0.5945, *p* = 0.5580); Two-way ANOVA, Tukey's multiple comparisons test; *p < 0.05. **b** Cochlear adiponectin levels were measured in the whole cochlear lysates. Dietary fat treatment increased cochlear adiponectin level only in female mice, but not in male mice. Two-way ANOVA, Fisher's LSD; ^a^main effect of sex, *F*_(1, 33)_ = 13.24, *p* = 0.0009; ^b^main effect of diet, *F*_(2, 33)_ = 4.303, *p* = 0.0218. **c** Serum/circulating leptin level showed a significant increase in HFD-fed mice compared to control diet. Two-way ANOVA; Tukey's multiple comparisons test; ^a^main effect of sex, *F*_(1, 28)_ = 2.437, *p* = 0.1298; ^b^main effect of diet, *F*_(2,28)_ = 17.81, *p* < 0.0001; *p < 0.05. **d** Cochlear leptin level showed no significant differences among groups. Main effect of sex, *F*_(1, 31)_ = 2.249, *p* = 0.1438; main effect of diet, *F*_(2,31)_ = 0.1752, *p* = 0.8401, *ns* not significant

Adiponectin plays pivotal roles as an otoprotectant against hearing loss and as a modulator of obesity-related metabolic disorders [9–12]. To investigate the association of adiponectin with hearing impairment and sexual dimorphism, the protein level of total adiponectin was measured in serum and cochlea by ELISA. As shown in Fig. 5a, a significant main effect of sex (*F*_1, 31_ = 55.00, *p* < 0.0001) was observed when comparing the circulating adiponectin levels between female and male mice in all diet groups. A main effect of diet was not observed for serum adiponectin (*F*_2, 31_ = 0.5945, *p* = 0.5580). An HFD significantly increased the cochlear adiponectin levels in female mice (10% vs*.* 45%, *p* = 0.0035; 10% vs*.* 60%, *p* = 0.0227), but not in male mice (Fig. 5b). Main effects of sex (*F*_1, 33_ = 13.24, *p* = 0.0009) and diet (*F*_2, 33_ = 4.303, *p* = 0.0218) were observed in the cochlear adiponectin levels. Therefore, the total expression of adiponectin shows sexual dimorphism, which may contribute to the enhanced protection against hearing loss seen in female mice.

### Leptin is regulated by diet but not sex

To investigate whether other adipokines contribute to the sex differences after HFD feeding, the serum and cochlear levels of leptin were measured by ELISA (Fig. 5c–d). A significant main effect of diet (*F*_2,28_ = 17.81, *p* < 0.0001) was observed when comparing the circulating leptin levels between female and male mice in all dietary groups. Local leptin levels in whole cochlear lysates exhibited no significant differences among the groups. These data support the idea that leptin is more affected by diet than sex, and is only weakly associated with hearing loss. Therefore, we focused on adiponectin.

### Expression of adiponectin and adiponectin receptor 1 in the inner ear

**Fig. 6:**
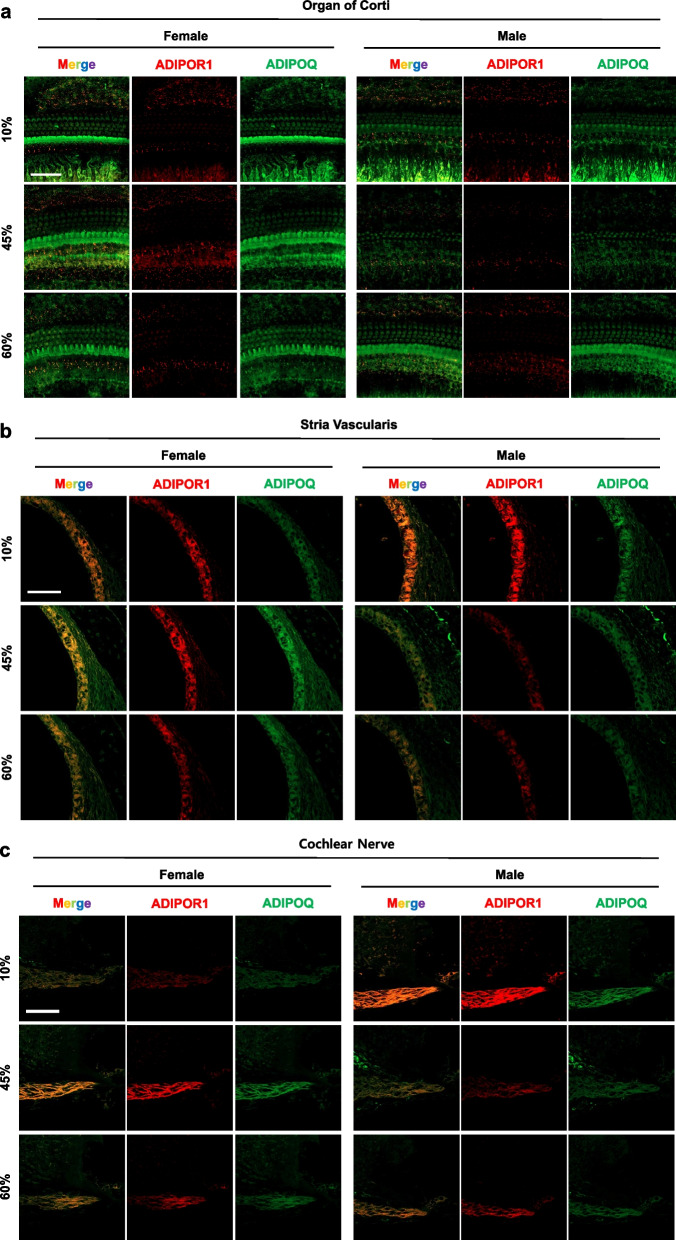

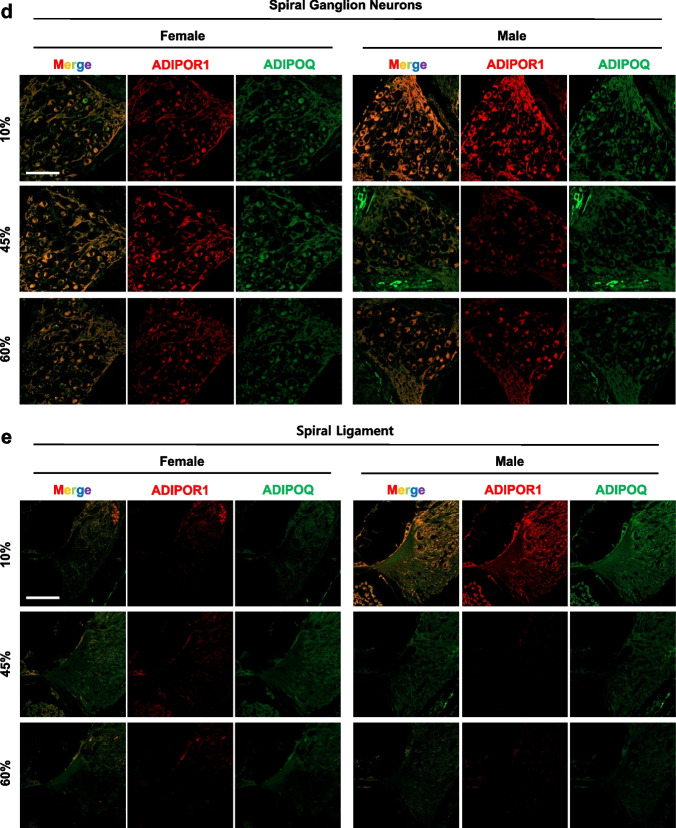
Expression of adiponectin and adiponectin receptor 1 in the inner ear. To visualize adiponectin receptor (red) and adiponectin (green), whole-mount of the auditory epithelium (**a**) or paraffin sections (**b**–**e**) of cochlea were prepared. Adiponectin receptors (red) were appeared as puncta in the inner hair cell membrane, Claudius cells, Hensen cells, inner phalangeal cells and cochlear nerve and neurons. Adiponectin (green) were expressed throughout the organ of Corti, mostly in the inner hair cells. Scale bar = 50 μm

Adiponectin (AdipoQ) and adiponectin receptor (AdipoR1) were visualized in the inner ear by immunostaining of whole-mount cochlea and paraffin sections (Fig. 6). In whole-mount organ of Corti, AdipoR1 was visualized as puncta in the membrane of supporting cells, and in the junction of the IHC and spiral ganglion neurons (Fig. 6a). In paraffin sections, AdipoR1 was highly expressed by supporting cells, as well as the cochlear nerve, spiral ganglion neurons, spiral ligaments, stria vascularis, and HCs. The expression level was highest in the supporting cells and cochlear nerve. Adiponectin was detected throughout the inner ear.

**Fig. 7:**
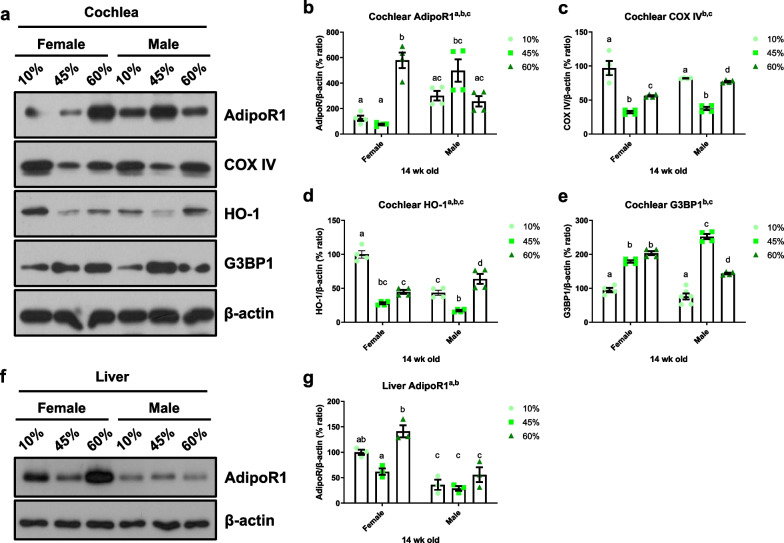
HFD regulates cochlear AdipoR1, COX IV, HO-1 and G3bp1; and liver AdipoR1. Cochlear AdipoR1, cytochrome *c* oxidase subunit IV (COX IV) heme oxygenase-1 (HO-1; also known as heat shock protein 32, HSP 32), and a marker of stress granule (G3bp1) levels were measured in the whole cochlear lysates by Western blot analysis (**a**) and quantified (**b**–**e**). Cochlear AdipoR1 and HO-1 presented main effects of sex and diet while COX IV and G3bp1 displayed a main effect of diet. **f** Liver AdipoR1 protein levels were measured by Western blotting (**a**) and quantified (**g**). Female AdipoR1 levels were regulated by diet, but no changes were seen in male AdipoR1 in the liver. Different letters indicate significant differences between treatments. Two-way ANOVA with Tukey’s multiple comparisons test. *p* < 0.05. ^a^main effect of sex; ^b^main effect of diet; ^c^interaction effect

Next, the cochlear protein levels of total AdipoR1 were quantified by Western blotting. As shown in Fig. 7, significant main effects of sex (*F*_1, 18_ = 6.642, *p* = 0.0190) and diet (*F*_2, 18_ = 9.980, *p* = 0.0012) were observed when comparing the cochlear AdipoR1 levels of female and male mice in all dietary groups. An HFD significantly increased the cochlear AdipoR1 levels in female mice (10% vs*.* 60%, *p* < 0.0001) but not male mice (10% vs. 45%, *p* = 0.0973; 10% vs. 60%, *p* = 0.9867), indicating that the female, but not male, cochlear AdipoR1 level is regulated by fat modulation.

To investigate the association between the local AdipoR1 level and the peripheral AdipoR1 level, total liver AdipoR1 was measured by Western blotting (Fig. 7f). Main effects of sex (*F*_1, 12_ = 61.10, *p* < 0.0001) and diet (*F*_2, 12_ = 15.70, *p* = 0.0004) were observed in terms of the liver AdipoR1 levels. Notably, the total AdipoR1 levels in male mice were significantly lower than in female mice in all diet groups and were not regulated by dietary modulation in males. Therefore, liver AdipoR1 expression shows sexual dimorphism, which may reflect the enhanced availability for adiponectin (a ligand) and protection against diet-induced obesity in female mice.

### HFD regulates cochlear COX IV and HO-1

An HFD can impair mitochondrial function, including mitochondrial dynamics and biogenesis [22]. To determine whether HFD can regulate the mitochondrial respiratory chain complexes, we measured the protein levels of cytochrome c oxidase subunit IV (COX IV), and heme oxygenase-1 (HO-1; also known as heat shock protein 32, HSP 32) in whole-cochlea lysates. Both HFD-fed female and male mice showed decreased COX IV levels (main effect of diet, *F*_2, 18_ = 77.05, *p* < 0.0001, Fig. 7a, c), whereas no significant sex differences were found (main effect of sex, *F*_1, 18_ = 1.064, *p* = 0.3159). The level of HO-1 was significantly decreased by the HFD (45% fat) in both female and male mice (Fig. 7d). These data suggest that mitochondrial function in the cochleae of both sexes is impaired by an HFD.

### Stress and inflammatory responses by HFD

To further investigate whether HFD regulates the local stress responses, a marker of stress granules (Ras GTPase-activating protein-binding protein 1, also known as GAP SH3 domain-binding protein 1) was quantified using an anti-G3BP1 antibody. Stress granules (SGs) are membrane-free cytosolic aggregations of RNA-binding proteins that form in response to stress and are essential for maintenance of cochlear function [23, 24]. Recent studies suggest that age-related stress granule dysfunction may be one of the main mechanisms underlying age-related hearing loss and neurodegenerative diseases [25]. An HFD significantly induced G3BP1 in male and female cochleae (main effect of diet, *F*_2, 20_ = 180.7, *p* < 0.0001, Fig. 7e); no significant sex differences were seen (main effect of sex, *F*_1, 20_ = 0.1117, *p* = 0.7417). These data suggest that a peripheral metabolic stress, such as that induced by an HFD, impacts the local stress responses regardless of sex, those responses may play a protective role in terms of cochlear function.

**Fig. 8:**
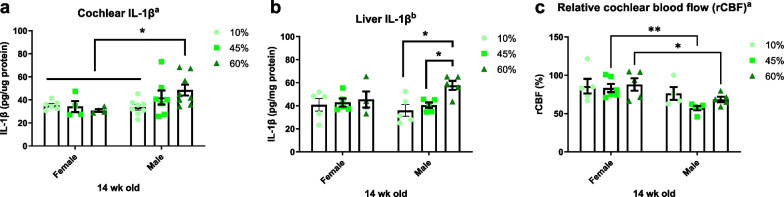
Local and periphery IL-1β and cochlear blood flow. **a** A proinflammatory cytokine, IL-1β, was quantified by ELISA in the whole cochlear lysates at 14 weeks of diet treatment. A significant main effect of sex (*F*_1, 31_ = 4.692, *p* = 0.0381, Two-way ANOVA), but no main effect of diet (*F*_2, 31_ = 0.7817, *p* = 0.4664) was observed. **b** Peripheral inflammation was measured in the liver IL-1β. A 60% fat diet increased IL-1β in male mice. Two-way ANOVA with Tukey’s multiple comparisons test. *p* < 0.05. a, main effect of sex; b, main effect of diet. **c** The cochlear blood flow was measured at 14 wks of age from male and female mice after feeding diets for 10 weeks. HFD-induced obesity impacts the cochlear microcirculation in the male mice. Two-way ANOVA with Fisher’s LSD. *p* < 0.05. ^a^main effect of sex; ^b^main effect of diet. *p < 0.05; **p < 0.01

Next, we quantified the levels of the proinflammatory cytokine interleukin 1 beta (IL-1β) in whole cochlear lysates via ELISA because an HFD has been reported to induce cochlear inflammation [26]. The IL-1β levels showed a significant main effect of sex (*F*_1, 31_ = 4.692, *p* = 0.0381, two-way ANOVA) but no main effect of diet (*F*_2, 31_ = 0.7817, *p* = 0.4664) after 14 weeks of dietary treatment (Fig. 8a). To further investigate whether local inflammation reflected peripheral changes, we measured liver IL-1β levels by ELISA. The 60% fat diet increased the IL-1β level in only male liver lysates, suggesting the IL-1β production is associated with the extent and phenotype of HFD-induced obesity. Increased peripheral IL-1β levels may affect the activation and production of cochlear IL-1β.

### Sex differences in cochlear blood flow

Finally, we measured cochlear blood flow in male and female mice at 14 weeks of age, to determine whether the inner ear is affected by the HFD-induced blood supply restriction. There was a significant sex difference (main effect of sex, *F*_1, 24_ = 11.52, *p* = 0.0024; and a main effect of diet, *F*_2, 24_ = 1.469, *p* = 0.2502) in cochlear blood flow (Fig. 8c), indicating that HFD-induced obesity affects the cochlear microcirculation.

## Discussion

Although male and female mice fed an HFD both showed body weight increase, several sex differences were observed. Male, but not female, mice exhibited greater weight gain, hyperglycemia, increased ABR thresholds at low frequencies and elevated DPOAE. The sexually dimorphic effect of HFD was also reflected in the amplitude of ABR wave 1, which correlated with CtBP2. The serum adiponectin concentration was significantly higher in female compared to male mice; the cochlear adiponectin levels were elevated by HFD in female, but not male mice. Adiponectin receptor 1 (AdipoR1) appeared as puncta in the IHC membrane, Claudius cells, Hensen cells, inner phalangeal cells, cochlear nerve, and neurons; AdipoR1 protein levels were increased by HFD in female, but not male, mice. Surprisingly, liver levels pf AdipoR1 were significantly lower in male than female mice in all dietary groups, supporting the possible otoprotective roles of local AdipoQ and cochlear AdipoR1. Stress granules (G3BP1) were significantly induced by an HFD in both sexes; conversely, inflammatory (IL-1β) responses were observed only in the male liver and cochlea.

Male B6.BKS(D)-*Lepr*^db^/J (db/db, diabetes) mice exhibited hearing impairment, microangiopathy, mitochondrial dysfunction, and synaptopathy in the inner ear, suggesting an association between obesity/type 2 diabetes and hearing impairment [4]. Yang and colleagues showed that DIO exacerbates hearing deficit in male CD-1 mice. In HFD-fed CD/1 mice, significant disruption of blood vessels in the stria vascularis was seen, along with an increased inflammatory response and cell loss/death in the basal turn of the cochlea due to activation of caspase-dependent and -independent apoptosis pathways [27]. Population studies have demonstrated an association between obesity and hearing impairment [15, 28, 29]. Hwang et al. conducted a cross-sectional study of the association between obesity and age-related hearing loss among the Taiwanese population; waist circumference, age, BMI, smoking, coronary artery disease and chronic renal failure were associated with hearing impairment [2]. Two studies of obesity and sudden sensorineural hearing loss (SSNHL) also found an association between obesity (defined as elevated total cholesterol and triglyceride levels, and a BMI > 27) and the prevalence of SSNHL [3, 30]. Kang et al. investigated hearing thresholds among South Koreans, including those with metabolic syndrome (MetS) and chronic kidney disease, and reported that men and women with MetS had higher hearing thresholds than those without [31]. Preclinical studies indicated a strong association between auditory function and obesity and/or diabetes in mouse (CD /1 [27] and CBA/CaJ [32] strains) and rat [33] models. In a streptozotocin injection-induced type 1 diabetes model, and in hyperlipidemic apolipoprotein E-knockout aging mice, HFD exacerbated hearing loss [32, 34, 35]. These data suggest that obesity-related metabolic alterations play a casual role in hearing impairment.

Sex differences have been reported in response to dietary fat manipulation [13, 36–39]. Based on the changes in body fat distribution during menopause, it was hypothesized that hormone replacement therapy reduced obesity in this group. The landmark Women’s Health Initiative (WHI) showed that a lack of estrogen increased adiposity, whereas hormone replacement therapy was associated with greater fat loss in postmenopausal women [40]. Similarly, preclinical studies have shown that estrogen treatment of ovariectomized adult female rats or mice reduce adiposity [41]. Other works have found that male and female mice fed an HFD gain significantly more weight than the sex-specific control groups; however, obese male mice show greater weight gain and metabolic changes (including hyperglycemia, hyperinsulinemia, hyperleptinemia, and hypercholesterolemia) than obese female mice [37]. These results indicate that estrogen plays pivotal roles in adiposity, which may contribute to the sex differences in DIO.

However, recent studies support the hypothesis that sex differences in DIO may stem from factors such as inflammation and brain synaptic function, in addition to ovarian estrogen [13, 42]. Pettersson et al*.* reported that protection from HFD in female mice is mediated by a large anti-inflammatory Treg cell population in intra-abdominal adipose tissue [13]. Lainez et al. demonstrated impaired inflammatory responses and hypothalamic function in male, but not female, mice. Microglial activation, peripheral macrophage infiltration, reduced levels of synaptic proteins, and fewer spines on GnRH neurons (that express gonadotropin-releasing hormone) were observed in the hypothalami of males; females were resistant to the increases in inflammatory cytokines and did not show changes in microglial morphology changes or monocyte-derived macrophage infiltration regardless of gonadal status [42]. We have shown that the levels of a proinflammatory cytokine (IL-1β) increased in the liver and cochlea (Fig. 8a, b). The main question posed by Fig. 8 is why IL-1β production was prevented in female mice. We speculate that the sexually dimorphic adiponectin expression in the periphery may enhance resistance to HFD-induced obesity in female mice, thus protecting against inflammatory responses in the cochlea, and hearing loss, in female mice.

Sex differences in hearing loss have been reported in a few preclinical studies. Young [14] and old [15, 16] male mice suffered more hearing loss than female mice. Aging rhesus monkeys showed a significant interaction effect of age and sex on ABR latencies [43]. Mice deficient in both ERβ and CYP19A1 (the latter gene encodes the aromatase that converts androgens to estrogens) exhibited an impaired response of the auditory system to acoustic trauma [44]. Sex is a critical biological variable with respect to metabolism and hearing sensitivity; however, few preclinical studies have investigated sex differences in obesity-related hearing loss. We have shown that both 45% and 60% HFDs induce more severe synaptopathy in the cochleae of male than female mice (Fig. 4e, f). These results support the idea that an HFD induces sex-specific differences in expression of CtBP2 puncta in the inner hair cells and synapses (Fig. 4e, f), correlating with the ABR wave I amplitudes (Fig. 2j).

Adiponectin exerts a variety of beneficial systemic effects, such as decreasing inflammation, promoting insulin sensitivity and cell survival, and conferring otoprotection. Hwang et al. observed a protective role of adiponectin against age-related hearing loss in a clinical cohort, i.e., there was a positive correlation between the plasma adiponectin level and peripheral hearing ability in Taiwanese males and females [11]. Tanigawa et al. showed that the circulating adiponectin level was significantly associated with the hearing threshold in healthy Japanese men [45]. A preclinical study using adiponectin-knockout mice detected a protective effect of adiponectin against hearing deficit—Adiponectin deficiency worsened age-related hearing loss in adiponectin-knockout male mice generated on a C57BL/6 background [45]. Anti-apoptotic effects of adiponectin have been reported in cochlear explant cultures [12]. Our data support an otoprotective effect of adiponectin based on the positive association between the circulating adiponectin level and hearing ability in male and female mice. The circulating adiponectin level was higher in healthy female than male Greek students [46]. The mechanism underlying the sex disparities in adiponectin was discussed by Nishizawa et al. Testosterone treatment reduced the circulating adiponectin level in castrated mice, which was linked to a significant improvement in insulin sensitivity [47]; this may explain the lower circulating adiponectin level and more profound metabolic alterations seen in male mice after HFD treatment (Figs. 1, 5a).

AdipoR1 is expressed in various regions of the inner ear. In the whole-mount preparation of organ of Corti in this study, puncta of AdipoR1 were observed in the IHC membrane, Claudius cells, Hensen’s cells, inner phalangeal cells, cochlear nerve, and neurons (Fig. 6c). In cochlea sections, AdipoR1 was distributed in the stria vascularis and spiral ligaments (Fig. 6b, e). Wu et al. evaluated the effects of the adiponectin type 1 receptor genotypes of 1682 volunteers on hearing sensitivity [12]. Holland et al. showed that adiponectin, which is associated with AdipoR1 and AdipoR2, significantly induces ceramidase activity and promotes the formation of an antiapoptotic metabolite, sphingosine-1-phosphate (S1P) [48]. AdipoR1 is a regulatory switch of docosahexaenoic acid (DHA) in photoreceptors. AdipoR1 ablation is responsible for DHA reduction and progressive photoreceptor degeneration in AdipoR1^−/−^ mice [49]. Adiponectin receptors are widely distributed in the mouse hypothalamus, brainstem, cortical neurons, and endothelial cells, as well as in whole brain and pituitary extracts [50]. Adiponectin receptors, including AdipoR1, AdipoR2 and calreticulin, are widely expressed in the cochlea [12] and may thus play critical roles in the auditory system. However, the regulation and biological effects of adiponectin receptors in the inner ear, including in diseased states, require further investigation.

The induction of HSPs in response to cellular stress is an important pro-survival mechanism [51]. HSP 32 catalyzes the conversion of heme to CO, biliverdin, and free iron [52]. Induction of HSP32/HO-1 promotes HC survival in response to noise [53], ototoxic drugs [54–56], and hyperthermia [57]. HSP32/HO-1 expression is regulated in a sex-dependent manner (Fig. 7d). Sex-dependent differential effects of HO-1 were detected in a mouse model [58]. Deletion of adipose tissue-specific *HO-1* induced hyperglycemia and insulinemia in female, but not male mice and caused a significant decrease in heme oxygenase activity and adiponectin concentration in adipose tissue. Therefore, HO-1 expression in adipose tissue may exert a greater protective effect in females than males [58]. In the cochlea, the level of HO-1 was significantly decreased by the HFD (45% fat) in both female and male mice (Fig. 7d), whereas conversely, the level was increased by the 60% fat diet. The underlying mechanisms remain unknown, however, it seems clear that the cochlea responds differently to the two fat levels. The susceptibility to hearing impairment induced by an HFD in male mice seems to be multi-factorial. (1) Lower peripheral (serum/liver) AdipoQ and AdipoR1 are strongly associated with the susceptibility to a HFD of male mice. It was quite astonishing to observe the sex differences in AdipoR1 expression in the liver: significantly lower AdipoR1 levels in male mice, which was not regulated by dietary fat at all. (2) Increased adiposity by an HFD enhanced the proinflammatory responses (e.g., IL-1β, Fig. XXX) in the periphery. (3) We speculate that metabolites, cytokines and/or immune cells (i.e., macrophages) infiltrate the cochlea (influx to cochlea through stria vascularis and endolymph) or cochlear immune cells (i.e., macrophages) are activated anyhow and produce local IL-1β (Fig. 8a). Although we found increased IL-1β levels in the cochlea, we still do not know whether the macrophages secreting IL-1β are cochlear resident or infiltrated from the periphery (or both). (4) It seems plausible that cochlear- “resident” (tissue-specific) AdipoQ and AdipoR1 exist and contribute to otoprotection against HFD-induced hearing impairment. While liver AdipoR1 was significantly lower in male mice, its expression was relatively high in the cochlea (Fig. 7a, f). These data support the putative otoprotective effect of local AdipoQ and AdipoR1.

We found that obese males had fewer ribbon synapses (CtBP2), suggesting a possible mechanism of the sex differences in sensorineural alterations after an HFD. Previous studies have shown that obesity impairs cognitive function [59]. Bocarsly et al. reported that early-stage obesity reduced the density of dendritic spines and decreased the expression of presynaptic and presynaptic markers in the perirhinal and prefrontal cortices [59, 60]. The overall decrease in the synaptic markers, and the difference in adiponectin level, strongly supports associations between sex differences and obesity-induced sensorineural impairment. Although it remains possible that higher adiponectin synthesis by females precedes otoprotection, it plausible that loss of synaptic markers (postsynaptic density and ribbon synapses) may reflect a decline in cochlear synaptic function, followed by hearing failure, in obese male mice. The extent to which sensorineural synaptic changes explain obesity-related hearing loss deserves further study.

This study had several limitations. First, it remains unknown as to whether the elevated adiponectin levels induced by HFD (Fig. 5b) are tissue-derived or periphery-derived, and how these changes occurred without affecting the circulating adiponectin level in female mice. The adiponectin oligomer level in cerebrospinal fluid (CSF) is different from that in serum. In CSF, about 80% of adiponectin is trimeric; the remaining 20% is a low-molecular-weight isoform that can cross the blood–brain barrier [50]. Further studies are needed to determine whether adiponectin oligomers can cross the blood–brain barrier, or whether the adiponectin detected by ELISA (Fig. 5b) and immunofluorescence (Fig. 6) is locally or peripherally produced. Second, we did not investigate the various subtypes of adiponectin receptors (i.e., AdipoR2); and did not measure receptor levels in organs other than the liver and cochlea. Although the AdipoR1 levels were low in all dietary groups (Fig. 7f, g), this does not exclude the possibility that AdipoR2 functions in the liver [61]. Also, we investigated AdipoR1 level only in the liver (to measure “peripheral” expression of the protein). Adiponectin receptors are differentially expressed in various organs and cell lines [62–64], indicating that other organs may contribute to adiponectin signaling. Finally, we explored the association between AdipoQ/AdipoR1 and hearing sensitivity, but not the potential causal effect of adiponectin in vivo or in vitro. Murohara reported that adiponectin supplementation via adenoviral vectors and the jugular vein reversed hearing impairment in 6-week-old wild-type and AdipoQ-KO mice aged 6 weeks by 8 weeks of age [45]. In vitro treatment with adiponectin (2.5 µg/mL) ameliorates gentamicin-induced cytotoxicity in cochlear explants [12]. Further studies are needed to determine the effects of exogenous AdipoQ and AdipoRs on hearing in LFD- and HFD-fed male and female mice.

### Perspectives and significance

Males are more vulnerable to HFD-induced metabolic alterations and hearing deficits. The sexual dimorphism in hearing ability is reflected in the lower amplitude of ABR wave 1 in HFD-fed male mice, which correlates with loss of CtBP2. The core stress granule component, G3BP1, was induced by HFD in the cochleae of male and female mice and significant sex differences were found in cochlear blood flow and IL-1β concentration. The level of an otoprotective adipokine, adiponectin, was significantly higher in female mice compared to male mice, and the cochlear adiponectin (AdipoQ) levels were elevated by HFD in females, but not male mice. Finally, cochlear and liver AdipoR1 expression presented significant sex differences and AdipoR1 were widely expressed in the inner ear of CBA/Ca mice. These changes may contribute to the sex differences in hearing sensitivity caused by HFD-induced obesity.

## Data Availability

The datasets generated and/or analyzed during the current study are available from the corresponding authors on reasonable request.

## References

[1] Hruby A, Hu FB (2015). The epidemiology of obesity: a big picture. Pharmacoeconomics.

[2] Hwang JH, Wu CC, Hsu CJ, Liu TC, Yang WS (2009). Association of central obesity with the severity and audiometric configurations of age-related hearing impairment. Obesity.

[3] Lee JS, Kim DH, Lee HJ, Kim HJ, Koo JW, Choi HG (2015). Lipid profiles and obesity as potential risk factors of sudden sensorineural hearing loss. PLoS ONE.

[4] Lyu A-R, Kim T-H, Shin S, Kim E-H, Yu Y, Gajbhiye A (2021). Hearing impairment in a mouse model of diabetes is associated with mitochondrial dysfunction, synaptopathy, and activation of the intrinsic apoptosis pathway. Int J Mol Sci.

[5] Dalton DS, Cruickshanks KJ, Klein R, Klein BE, Wiley TL (1998). Association of NIDDM and hearing loss. Diabetes Care.

[6] Austin DF, Konrad-Martin D, Griest S, McMillan GP, McDermott D, Fausti S (2009). Diabetes-related changes in hearing. Laryngoscope.

[7] Cullen JR, Cinnamond MJ (1993). Hearing loss in diabetics. J Laryngol Otol.

[8] Dhanda N, Taheri S (2017). A narrative review of obesity and hearing loss. Int J Obes (Lond).

[9] Ouchi N, Parker JL, Lugus JJ, Walsh K (2011). Adipokines in inflammation and metabolic disease. Nat Rev Immunol.

[10] Shibata R, Murohara T, Ouchi N (2012). Protective role of adiponectin in cardiovascular disease. Curr Med Chem.

[11] Hwang JH, Hsu CJ, Liu TC, Yang WS (2011). Association of plasma adiponectin levels with hearing thresholds in adults. Clin Endocrinol.

[12] Wu C-C, Tsai C-H, Lu Y-C, Lin H-C, Hwang J-H, Lin Y-H (2015). Contribution of adiponectin and its type 1 receptor to age-related hearing impairment. Neurobiol Aging.

[13] Pettersson US, Waldén TB, Carlsson P-O, Jansson L, Phillipson M (2012). Female mice are protected against high-fat diet induced metabolic syndrome and increase the regulatory T cell population in adipose tissue. PLoS ONE.

[14] Kobrina A, Dent ML (2016). The effects of aging and sex on detection of ultrasonic vocalizations by adult CBA/CaJ mice (Mus musculus). Hear Res.

[15] Guimaraes P, Zhu X, Cannon T, Kim S, Frisina RD (2004). Sex differences in distortion product otoacoustic emissions as a function of age in CBA mice. Hear Res.

[16] Henry KR (2004). Males lose hearing earlier in mouse models of late-onset age-related hearing loss; females lose hearing earlier in mouse models of early-onset hearing loss. Hear Res.

[17] Lyu AR, Kim TH, Park SJ, Shin SA, Jeong SH, Yu Y (2020). Mitochondrial damage and necroptosis in aging cochlea. Int J Mol Sci.

[18] Ison JR, Allen PD, O’Neill WE (2007). Age-related hearing loss in C57BL/6J mice has both frequency-specific and non-frequency-specific components that produce a hyperacusis-like exaggeration of the acoustic startle reflex. J Assoc Res Otolaryngol.

[19] Liu H, Li G, Lu J, Gao Y-G, Song L, Li G-L (2019). Cellular differences in the cochlea of CBA and B6 mice may underlie their difference in susceptibility to hearing loss. Front Cell Neurosci.

[20] Speakman JR. Use of high-fat diets to study rodent obesity as a model of human obesity. Nature Publishing Group; 2019. p. 1491–2. 10.1038/s41366-019-0363-7 30967607

[21] Kujawa SG, Liberman MC (2015). Synaptopathy in the noise-exposed and aging cochlea: primary neural degeneration in acquired sensorineural hearing loss. Hear Res.

[22] Chen D, Li X, Zhang L, Zhu M, Gao L (2018). A high-fat diet impairs mitochondrial biogenesis, mitochondrial dynamics, and the respiratory chain complex in rat myocardial tissues. J Cell Biochem.

[23] Martin JL, Dawson SJ, Gale JE (2022). An emerging role for stress granules in neurodegenerative disease and hearing loss. Hear Res.

[24] Anderson P, Kedersha N (2006). RNA granules. J Cell Biol.

[25] Shen Y, Ye B, Chen P, Wang Q, Fan C, Shu Y (2018). Cognitive decline, dementia, Alzheimer's disease and presbycusis: examination of the possible molecular mechanism. Front Neurosci.

[26] Chan J, Telang R, Kociszewska D, Thorne PR, Vlajkovic SM (2022). A high-fat diet induces low-grade cochlear inflammation in CD-1 mice. Int J Mol Sci.

[27] Hwang J-H, Hsu C-J, Yu W-H, Liu T-C, Yang W-S (2013). Diet-induced obesity exacerbates auditory degeneration via hypoxia, inflammation, and apoptosis signaling pathways in CD/1 mice. PLoS ONE.

[28] Park Y-H (2021). Do women have better hearing than men?. Clin Exp Otorhinolaryngol.

[29] Villavisanis DF, Berson ER, Lauer AM, Cosetti MK, Schrode KM (2020). Sex-based differences in hearing loss: perspectives from non-clinical research to clinical outcomes. Otol Neurotol.

[30] Ballesteros F, Tassies D, Reverter J, Alobid I, Bernal-Sprekelsen M (2012). Idiopathic sudden sensorineural hearing loss: classic cardiovascular and new genetic risk factors. Audiol Neurotol.

[31] Kang SH, Jung DJ, Cho KH, Park JW, Yoon KW, Do JY (2015). The association between metabolic syndrome or chronic kidney disease and hearing thresholds in Koreans: the Korean National Health and Nutrition Examination Survey 2009–2012. PLoS ONE.

[32] Vasilyeva ON, Frisina ST, Zhu X, Walton JP, Frisina RD (2009). Interactions of hearing loss and diabetes mellitus in the middle age CBA/CaJ mouse model of presbycusis. Hear Res.

[33] McQueen CT, Baxter A, Smith TL, Raynor E, Yoon SM, Prazma J (1999). Non-insulin-dependent diabetic microangiopathy in the inner ear. J Laryngol Otol.

[34] Ishikawa T, Naito Y, Taniguchi K (1995). Hearing impairment in WBN/Kob rats with spontaneous diabetes mellitus. Diabetologia.

[35] Guo Y, Zhang C, Du X, Nair U, Yoo T-J (2005). Morphological and functional alterations of the cochlea in apolipoprotein E gene deficient mice. Hear Res.

[36] Casimiro I, Stull ND, Tersey SA, Mirmira RG (2021). Phenotypic sexual dimorphism in response to dietary fat manipulation in C57BL/6J mice. J Diabetes Complicat.

[37] Hwang LL, Wang CH, Li TL, Chang SD, Lin LC, Chen CP (2010). Sex differences in high-fat diet-induced obesity, metabolic alterations and learning, and synaptic plasticity deficits in mice. Obesity.

[38] Klein SL, Flanagan KL (2016). Sex differences in immune responses. Nat Rev Immunol.

[39] Palmer BF, Clegg DJ (2015). The sexual dimorphism of obesity. Mol Cell Endocrinol.

[40] Chmouliovsky L, Habicht F, James RW, Lehmann T, Campana A, Golay A (1999). Beneficial effect of hormone replacement therapy on weight loss in obese menopausal women. Maturitas.

[41] Grove KL, Fried SK, Greenberg AS, Xiao XQ, Clegg DJ (2010). A microarray analysis of sexual dimorphism of adipose tissues in high-fat-diet-induced obese mice. Int J Obes (Lond).

[42] Lainez NM, Jonak CR, Nair MG, Ethell IM, Wilson EH, Carson MJ (2018). Diet-induced obesity elicits macrophage infiltration and reduction in spine density in the hypothalami of male but not female mice. Front Immunol.

[43] Fowler CG, Torre P, Kemnitz JW (2002). Effects of caloric restriction and aging on the auditory function of rhesus monkeys (*Macaca mulatta*): The University of Wisconsin Study. Hear Res.

[44] Meltser I, Tahera Y, Simpson E, Hultcrantz M, Charitidi K, Gustafsson JA (2008). Estrogen receptor beta protects against acoustic trauma in mice. J Clin Investig.

[45] Tanigawa T, Shibata R, Ouchi N, Kondo K, Ishii M, Katahira N (2014). Adiponectin deficiency exacerbates age-related hearing impairment. Cell Death Dis.

[46] Yannakoulia M, Yiannakouris N, Bluher S, Matalas AL, Klimis-Zacas D, Mantzoros CS (2003). Body fat mass and macronutrient intake in relation to circulating soluble leptin receptor, free leptin index, adiponectin, and resistin concentrations in healthy humans. J Clin Endocrinol Metab.

[47] Nishizawa H, Shimomura I, Kishida K, Maeda N, Kuriyama H, Nagaretani H (2002). Androgens decrease plasma adiponectin, an insulin-sensitizing adipocyte-derived protein. Diabetes.

[48] Holland WL, Miller RA, Wang ZV, Sun K, Barth BM, Bui HH (2011). Receptor-mediated activation of ceramidase activity initiates the pleiotropic actions of adiponectin. Nat Med.

[49] Rice DS, Calandria JM, Gordon WC, Jun B, Zhou Y, Gelfman CM (2015). Adiponectin receptor 1 conserves docosahexaenoic acid and promotes photoreceptor cell survival. Nat Commun.

[50] Thundyil J, Pavlovski D, Sobey CG, Arumugam TV (2012). Adiponectin receptor signalling in the brain. Br J Pharmacol.

[51] Morimoto RI, Santoro MG (1998). Stress-inducible responses and heat shock proteins: new pharmacologic targets for cytoprotection. Nat Biotechnol.

[52] Maines MD, Trakshel GM (1992). Differential regulation of heme oxygenase isozymes by Sn- and Zn-protoporphyrins: possible relevance to suppression of hyperbilirubinemia. Biochim Biophys Acta.

[53] Matsunobu T, Satoh Y, Ogawa K, Shiotani A (2009). Heme oxygenase-1 expression in the guinea pig cochlea induced by intense noise stimulation. Acta Otolaryngol Suppl.

[54] Fernandez K, Spielbauer KK, Rusheen A, Wang L, Baker TG, Eyles S (2020). Lovastatin protects against cisplatin-induced hearing loss in mice. Hear Res.

[55] Francis S, Kramarenko I, Brandon C, Lee F, Baker T, Cunningham L (2011). Celastrol inhibits aminoglycoside-induced ototoxicity via heat shock protein 32. Cell Death Dis.

[56] Baker TG, Roy S, Brandon CS, Kramarenko IK, Francis SP, Taleb M (2015). Heat shock protein-mediated protection against cisplatin-induced hair cell death. J Assoc Res Otolaryngol.

[57] Fairfield DA, Kanicki AC, Lomax MI, Altschuler RA (2004). Induction of heat shock protein 32 (Hsp32) in the rat cochlea following hyperthermia. Hear Res.

[58] Hosick PA, Weeks MF, Hankins MW, Moore KH, Stec DE (2017). Sex-dependent effects of HO-1 deletion from adipocytes in mice. Int J Mol Sci.

[59] Bocarsly ME, Fasolino M, Kane GA, LaMarca EA, Kirschen GW, Karatsoreos IN (2015). Obesity diminishes synaptic markers, alters microglial morphology, and impairs cognitive function. Proc Natl Acad Sci USA.

[60] Martinez-Pena YVI, Akaaboune M (2020). The disassembly of the neuromuscular synapse in high-fat diet-induced obese male mice. Mol Metab..

[61] Alzahrani B, Iseli T, Ramezani-Moghadam M, Ho V, Wankell M, Sun EJ (2018). The role of AdipoR1 and AdipoR2 in liver fibrosis. Biochim Biophys Acta Mol Basis Dis.

[62] Jasinski-Bergner S, Buttner M, Quandt D, Seliger B, Kielstein H (2017). Adiponectin and its receptors are differentially expressed in human tissues and cell lines of distinct origin. Obes Facts.

[63] Sluch VM, Banks A, Li H, Crowley MA, Davis V, Xiang C (2018). ADIPOR1 is essential for vision and its RPE expression is lost in the Mfrprd6 mouse. Sci Rep.

[64] Parker-Duffen JL, Nakamura K, Silver M, Zuriaga MA, MacLauchlan S, Aprahamian TR (2014). Divergent roles for adiponectin receptor 1 (AdipoR1) and AdipoR2 in mediating revascularization and metabolic dysfunction in vivo. J Biol Chem.

